# Application of a selective hospitalization model in the clinical practice of breast surgery

**DOI:** 10.1097/MD.0000000000034209

**Published:** 2023-06-30

**Authors:** Ying Chen, Liying Huang, Xia Chen

**Affiliations:** a Department of Breast Surgery, Clinical Oncology School of Fujian Medical University, Fujian Cancer Hospital, Fujian, China.

**Keywords:** breast cancer, breast disease, length of hospital stay, medical expense, selective hospitalization model

## Abstract

This study aimed to explore the clinical application of a selective hospitalization model in breast disease specialties and to evaluate its effectiveness. Information of patients registered in the selective hospitalization model and those registered in the direct model between October 1, 2020, and October 31, 2022, were collected. The hospitalization days and expenses of patients admitted through distinct modes and divergent medical categories were examined. After completing relevant examinations during the selected hospitalization period, 708 patients were admitted to our medical group for further treatment during the study period. Furthermore, 401 patients underwent hospitalization procedures immediately after the initial visit and received additional treatment after completing pertinent examinations during hospitalization. For patients who underwent benign surgery after admission, there was a significant difference in the length of hospital stay between patients admitted through selective hospitalization and those admitted directly (*P* < .001); however, there was no significant difference in total hospital expenses (*P* = .895). For patients who underwent malignant surgery after admission, there were significant differences in the length of hospital stay (*P* < .001) and total cost of hospitalization (*P* = .015). There was no significant difference in the length of hospital stay between the 2 groups of patients initially admitted for neoadjuvant chemotherapy (*P* = .589); however, the total cost of hospitalization significantly differed (*P* < .001). The selective hospitalization model can reduce medical expenses and the average length of hospital stay. This new hospitalization model is more flexible and allows for the inclusion of outpatient examination costs in subsequent hospitalization medical insurance reimbursement, greatly reducing the financial burden on patients. It is worthy of further exploration, optimization, and promotion.

## 1. Introduction

Cancer is an onerous burden in both developed and developing countries.^[1]^ According to the statistics of “GLOBOCAN,” there were approximately 19,292,789 new cancer cases and 9958,133 cancer-related deaths, worldwide, in 2020.^[2]^ Furthermore, breast cancer has been identified as the most prevalent malignancy and the leading cause of cancer-related death in women worldwide^[3]^ and in China.^[4]^ It has become a serious threat to public health and has led to an escalation in medical costs, posing an economic burden^[5]^ on patients and families and leading to severe social inequity. To improve access to healthcare, the Chinese government has made a notable commitment to establish Social Health Insurance in the last 3 decades, and many efforts have been made to further improve the benefits package of the financial protection provided by the insurance.^[6,7]^ However, regardless of the type of insurance, most examination expenses incurred by outpatients before a cancer diagnosis are not included in the reimbursement scope of Social Health Insurance. To solve this problem and reduce the burden on patients, the Fujian Cancer Hospital designed and implemented a new hospitalization model, namely selective hospitalization model.

The selective hospitalization model is used predominantly for nonemergency surgical patients who satisfy the surgical criteria for hospitalization via a comprehensive evaluation, as well as radiotherapy and chemotherapy patients who need to be hospitalized. After a comprehensive evaluation by the attending physicians, the patient is identified as being eligible for selective hospitalization and undergoes an appropriate pre-hospitalization examination in the outpatient department. On completion of the examination, the patient is transferred to a conventional hospital. Distinct from a simple outpatient visit, the outpatient expenses during selective hospitalization are included in the hospitalization expense reimbursement from the medical insurance. In particular, the outpatient inspection and treatment expenses incurred in the same designated medical institution as part of the selective hospitalization procedure can be included in the medical insurance hospitalization expense claim for settlement within 14 days of completing the selective hospitalization registration procedure.

The Breast Surgery Department at Fujian Cancer Hospital, where the author works, has engaged in selective hospitalization for patients admitted to the department. The department became familiar with the handling procedure gradually, through early pilot testing and began promoting it comprehensively in October 2020. To better explain the benefits of this model for hospitalized patients, we analyzed the patients admitted to the hospital in the selective hospitalization model and those registered in the direct model and compared the length of hospital stay, hospitalization expenses, and other key indicators. We also evaluated the value of selective hospitalization in clinical practice.

## 2. Materials and methods

### 2.1. General information

The author’s diagnosis and treatment group began registering the information of patients who underwent selective hospitalization and those admitted in the direct model to the group on October 1, 2020. As of October 31, 2022, 1109 patients who were first hospitalized for treatment were selected from the data and subdivided according to whether they underwent selective hospitalization or direct hospitalization, and the differential treatment measures implemented after hospitalization were collected. Furthermore, 29 patients with severe diseases were transferred to other departments for further diagnosis and treatment.

### 2.2. Research methods

This study was approved by the Ethics Committee of the Fujian Cancer Hospital. Written informed consent was obtained from all the patients. The patients’ medical records, including their outpatient medical records, outpatient registration times, hospitalization times, discharge times, waiting times, hospitalization days, inpatient medical records, diagnoses and treatment categories, hospitalization expenses, and other data, were collected. Indicators such as the hospitalization days and the expenses of patients admitted through different ways and the divergent diagnoses and treatment categories were analyzed.

### 2.3. Statistical analysis

The statistical analysis was performed using SPSS 26.0 (IBM Corp, IBM SPSS Statistics for Windows, Version 26.0. Armonk, NY), and the measurement data were expressed as the means (minimum-maximum). The Student *t* test was used for the significance analysis. The statistical significance was set at *P* < .05.

## 3. Results

### 3.1. General information

Between October 1, 2020, and October 31, 2022, 708 patients were registered in the authors’ diagnosis and treatment group in the selective hospitalization model and finally admitted to our group for formal hospitalization and further treatment after completing the relevant examinations in the outpatient department, and 29 patients were registered in our group, but transferred to other departments for further diagnosis and treatment because of other findings during the outpatient examination period. On the other hand, 401 patients underwent hospitalization immediately after the first outpatient visit and received treatment after completing pertinent examinations during hospitalization. Based on the divergent diagnoses and treatment measures taken after hospitalization, the patients were subdivided into benign surgery, malignant surgery, and first-time neoadjuvant chemotherapy groups. The specific distributions of the patients are presented in Table 1.

**Table 1:** Distribution of patients admitted to the hospital by varying routes and the distinct treatment measures taken after hospitalization.

Admission route Treatment measure	Benign surgery	Malignant surgery	First-time neoadjuvant chemotherapy	No treatment	Total
Selective hospitalization	328	240	128	12	708
Directly hospitalization	168	173	43	17	401
Total	496	413	171	29	1109

Distinct categories of treatment measures have varying impacts on corresponding hospitalization days and expenses; therefore, further analysis was performed based on the categories of treatment measures. The results are presented in Tables 2 and 3.

**Table 2:** Distribution of hospital stay by varying routes and the distinct treatment measures taken after hospitalization (days).

Admission route Treatment measure	Benign surgery	Malignant surgery	First-time neoadjuvant chemotherapy
Selective hospitalization	3.34 (1–17)	8.97 (5–22)	6.64 (2–19)
Directly hospitalization	5.20 (2–16)	11.60 (6–26)	7.07 (2–19)
*P* value	*P* < .001	*P* < .001	*P* = .589

**Table 3:** Distribution of total cost of hospitalization by varying routes and the distinct treatment measures taken after hospitalization (CNY).

Admission route Treatment measure	Benign surgery	Malignant surgery	First-time neoadjuvant chemotherapy
Selective hospitalization	5687.68 (2172.43–17123.57)	24811.00 (12636.53–80282.85)	28579.04 (11851.02–52841.60)
Directly hospitalization	5710.00 (3076.77–13,233.10)	23143.05 (9311.08–64243.04)	19519.72 (5864.83–42544.90)
*P* value	*P* = .895	*P* = .015	*P* < .001

CNY = Chinese Yuan.

### 3.2. Comparison of patients admitted for benign surgery

**Figure 1.**
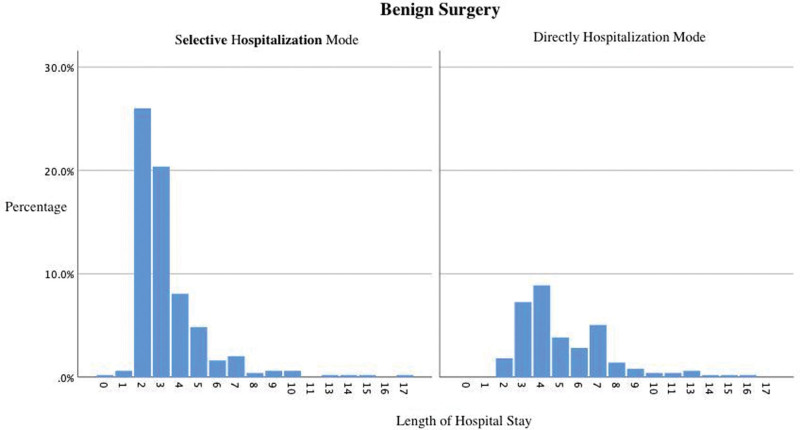
Distribution of hospital days for patients who underwent benign surgery after admission.

Among the patients who underwent benign surgery after admission, 328 were admitted in the selective model. The average length of hospital stay was 3.34 days (1–17 days), and the average total cost of hospitalization was 5687.68 Chinese Yuan (CNY) (2172.43–17123.57 CNY). On the other hand, the total number of patients admitted immediately to the hospital for benign surgery was 168, the average length of hospital stay was 5.20 days (2–16 days), and the average total cost of hospitalization was 5710.00 CNY (3076.77–13,233.10 CNY). There was a significant difference in the length of hospital stay (as shown in Fig. 1) between the divergent admission routes (*P* < .001); however, there was no significant difference in the total cost of hospitalization between the 2 groups (*P* = .895).

### 3.3. Comparison of patients admitted for malignant surgery

**Figure 2.**
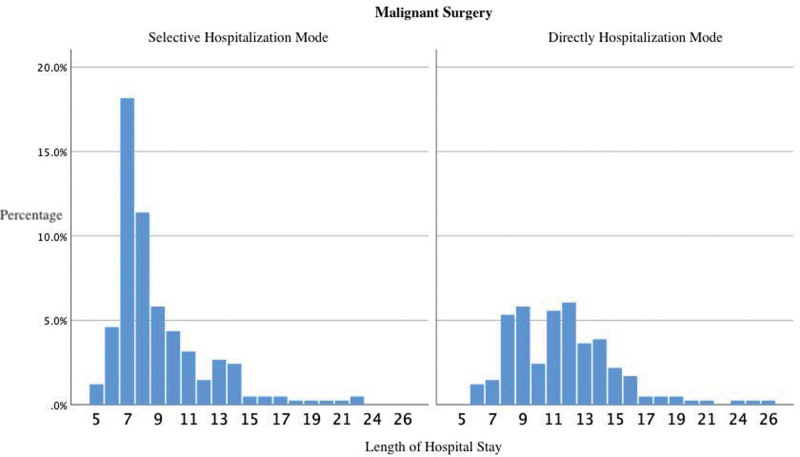
Distribution of hospital days for patients who underwent malignant surgery after admission.

The total number of patients admitted in the selective model who underwent malignant surgery after admission was 240, the average length of hospital stay was 8.97 days (5–22 days), and the average total cost of hospitalization was 24811.00 CNY (12636.53–80282.85 CNY). Furthermore, the total number of patients admitted directly to the hospital for malignant surgery was 173, the average length of hospital stay was 11.60 days (6–26 days), and the average total cost of hospitalization was 23143.05 CNY (9311.08–64243.04 CNY). There was a significant difference in the length of hospital stay (as shown in Fig. 2) and total cost of hospitalization between the different admission routes (*P* < .001; *P* = .015).

### 3.4. Comparison of patients admitted for first-time neoadjuvant chemotherapy

**Figure 3.**
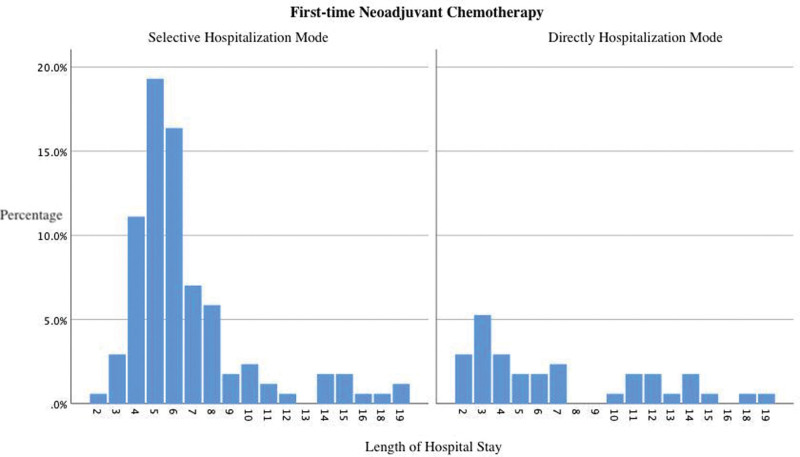
Distribution of hospital days for patients who underwent first-time neoadjuvant chemotherapy after admission.

Among the patients admitted to the hospital for first-time neoadjuvant chemotherapy, 128 were admitted in the selective model. The average length of hospital stay was 6.64 days (2–19 days), and the average total cost of hospitalization was 28579.04 CNY (11851.02–52841.60 CNY). Forty-three patients received first-time neoadjuvant chemotherapy after being admitted directly to the hospital to complete relevant examinations. The average length of hospital stay was 7.07 days (2–19 days), and the average total cost of hospitalization was 19519.72 CNY (5864.83–42544.90 CNY). There was no statistically significant difference in the length of hospital stay between patients admitted in the selective model and those admitted directly (*P* = .589). However, from the distribution chart of the length of hospital stay (as shown in Fig. 3), we observed that the length of hospitalization for selectively admitted patients was relatively condensed over a shorter period. There was a significant difference in the total cost of hospitalization between the 2 groups (*P* < .001).

### 3.5. Comparison of patients who ultimately do not require treatment after examination

**Figure 4.**
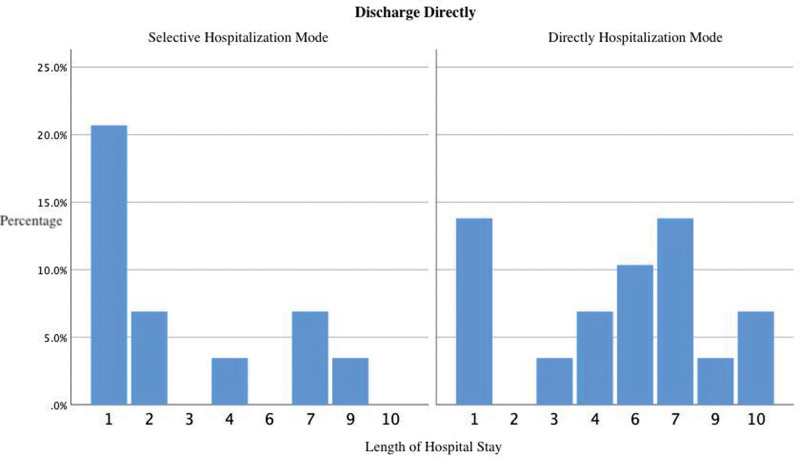
Distribution of hospital days for patients who need no further treatment and directly go through discharge procedures.

In addition, a few patients require no further treatment after completing the relevant examinations and undergo direct discharge procedures for various reasons. There was no significant difference (*P* = .064) in the length of hospital stay (as shown in Fig. 4) between patients admitted in the selective model (n = 12, 3.08 days [1–9 days]) and those admitted immediately (n = 17, 5.29 days [1–10 days]). There was also no significant difference (*P* = .626) in the total cost of hospitalization between the 2 groups (5310.54 CNY [228.00–17058.47 CNY] vs 4645.62 CNY [171.00–10146.60 CNY]).

### 3.6. Patients transferred to other departments

We also focused on 29 patients who were registered in the author’s outpatient but were transferred to other departments for further treatment after examination. These patients were often initially diagnosed with breast-related diseases and hoped to receive the corresponding treatment in the surgery department. However, other severe diseases were found during the examination. Two of the 29 patients had relatively mild breast disease but were diagnosed with thyroid cancer (1 case) or cervical cancer (1 case) and transferred to the appropriate specialty. The remaining 27 patients were diagnosed with initial stage IV breast cancer due to the discovery of distant metastasis after examination and were transferred to the medical oncology department for palliative care.

### 3.7. Waiting time before hospitalization

Corresponding statistics were calculated on the waiting days before hospitalization for patients admitted in the selective model. The average waiting days were 6.65 days (0–48 days) for patients who underwent benign surgery after admission, 7.90 days (0–31 days) for those who underwent malignant surgery after admission, 9.51 days (2–21 days) for those who received first-time neoadjuvant chemotherapy, and 7.83 days (1–25 days) for those who were admitted but did not receive any treatment.

## 4. Discussion

As people’s economic status improves and they become more health conscious, more women seek medical advice on breast disease. Among breast diseases, malignant tumors are the most feared. According to the statistical data of the Fujian cancer registration system^[8]^ of 2021, the most common malignant tumor in women was breast cancer, accounting for 42.36/10^5^ and 51.14/10^5^ of women in urban areas, respectively. After gaining awareness of their abnormalities, patients often seek further diagnosis and treatment in hospitals. However, in clinical practice, patients visiting physicians must undergo pertinent examinations to develop treatment plans. If all relevant examinations are completed during hospitalization, this will inevitably lead to a longer hospital stay and a decrease in bed turnover rate.^[9]^ However, if completed within the outpatient department, patients face the problem of expenses incurred in the outpatient department not being covered by health insurance reimbursement, increasing the burden on patients.

The selective hospitalization model is a new model for patients to complete related examinations before actually checking into the ward. Although all of the examinations are completed within the outpatient department, the expenses can be included in the hospitalization expense reimbursement from the medical insurance. This new hospitalization model is more flexible and can greatly reduce the financial burden on patients, as our study results showed above.

Based on the analysis of this study’s data in the past 2 years, we discovered that among patients who underwent benign surgery after admission, the average hospitalization days for those who opted for the selective model were shortened by approximately 2 days. From the distribution chart of hospitalization days (Fig. 1), we observed that most hospitalization days fall between 2 and 4 days, and there was no significant difference in hospitalization costs between the 2 groups. Furthermore, we are exploring the mode of daytime surgery for patients undergoing benign surgery^[10,11]^; however, the actual development of this mode requires the efficient cooperation of the entire team of doctors, ward nurses, operating rooms, pathology departments, and patients^[12]^ and is still in the exploratory phase. Before this model matures, selective hospitalization can solve the problems of hospitalization surgery and related medical insurance reimbursements for these patients.

The average length of hospital stay was reduced by approximately 3 days for patients who underwent malignant surgery at admission in the selective hospitalization group. Similarly, from the distribution chart of hospitalization days (Fig. 2), we observed that the length of hospitalization was predominantly concentrated in the shorter 6–9 days. The waiting time before surgery should be shortened as much as possible to provide more space for patients to recover and recuperate postoperatively, which will be convenient for doctors to observe the situation postoperatively. For patients with breast cancer, physicians need to pay more attention to the precise preoperative location of the mass or even nonpalpable breast cancer^[13]^ and postoperative wound complications, especially the prevention and treatment of seroma^[14,15]^ after axillary dissection. The total cost of hospitalization was slightly higher for patients admitted in the selective model. However, in clinical practice, most patients directly admitted for malignant surgery have undergone relevant examinations in other hospitals before admission, and some were transferred to our hospital after being diagnosed with cancer in other hospitals. When most people are still “turning pale about cancer,” it may be more appropriate for these patients to be admitted directly for their psychological comfort and protection. Consequently, expenses incurred after admission diminish after the relevant examination have done in the external hospital.

For patients admitted for first-time neoadjuvant treatment, the difference in the length of hospital stay between the 2 groups was insignificant. However, the waiting time before admission for patients admitted in the selective model was the longest, with an average of 9.51 days, which was related to the completion of breast and/or axillary core needle biopsies during the outpatient examination. According to the National Comprehensive Cancer Network Guidelines and Guidelines and Specifications for the Diagnosis and Treatment of Breast Cancer of the Chinese Anti-Cancer Association (2021 Edition),^[16]^ among patients with locally advanced inoperable breast cancer, and those who subjectively strongly require breast conservation after the descending stage as well as those with Her2-positive or triple-negative breast cancer with a certain tumor load have indications for preoperative therapy. Before neoadjuvant treatment, determining the tumor stage is essential because the therapeutic schedule depends on the pathological type and molecular classification. Patients are also required to obtain pathology and subsequent further analysis by the pathologist through biopsy before the start of neoadjuvant treatment. This process requires a certain amount of waiting time. For patients with Her2++, as indicated by the immunohistochemical results, further fish gene detection is required to determine the amplification of the Her2 gene.^[17]^ If the above process can be performed in the outpatient department and included in the medical insurance reimbursement in the subsequent hospitalization process, patients’ anxiety and discomfort will be significantly alleviated, the impact of the hospital’s busy environment during the waiting process will be avoided, and patients can receive treatment faster when needed. Furthermore, most patients directly admitted to the hospital for neoadjuvant treatment were transferred to our hospital after being diagnosed at an external hospital, and treatment was initiated immediately. Therefore, there was no significant difference in the length of hospital stay between the 2 groups. However, patients directly admitted to the hospital incurred significantly lower hospitalization costs. For those patients, most examinations in external hospitals are completed in the outpatient department; therefore, they are excluded from medical insurance reimbursements. For individual patients, the accumulated expenditure is greater.

In addition, a few patients do not require further treatment after completing the relevant examinations and undergo direct discharge procedures for various reasons. No significant differences are observed between those who are selectively admitted and those who are not; therefore, the formalities do not increase the burden on these patients. According to an analysis of previous patient types, patients experience greater benefits if they receive additional treatment later.

Patients newly diagnosed with stage IV breast cancer in whom other lesions or distant metastases are discovered during the outpatient examination in selective model will be transferred directly to the corresponding specialty for further treatment under the circumstances of selective hospitalization, making the transfer of patients more flexible, reducing repeated examinations and unnecessary admissions, achieving optimal utilization of medical and health resources, and further improving the hospitalization turnover rate.

## 5. Conclusions

This study’s findings demonstrate the benefits of selective hospitalization. Applying selective hospitalization in clinical practice demonstrates its extraordinary contribution to lowering medical expenses and reducing the average length of hospital stay. Selective hospitalization is more flexible, allowing patients to clearly understand the type of disease and related diagnosis and treatment plans before admission and enabling them to make timely adjustments based on different conditions. Furthermore, this portion of outpatient expenses can be included in subsequent hospitalization medical insurance reimbursements, greatly reducing the financial burden on patients. We will apply this model in future clinical practice and constantly improve it to bring greater convenience to patients and reduce the economic burden and mental distress caused by the disease.

## Author contributions

**Data curation:** Ying Chen.

**Investigation:** Liying Huang.

**Project administration:** Liying Huang.

**Writing – original draft:** Ying Chen.

**Writing – review & editing:** Xia Chen.
